# Improving the Early Assessment of Child Neglect Signs—A New Technique for Professionals

**DOI:** 10.3390/pediatric15020035

**Published:** 2023-06-19

**Authors:** Giovanni Giulio Valtolina, Concetta Polizzi, Giovanna Perricone

**Affiliations:** 1 Italian Society of Pediatric Psychology (S.I.P.Ped.), 90121 Palermo, Italy; 2 Department of Psychology, Catholic University of the Sacred Heart, 20121 Milan, Italy; 3 Department of Psychology, Educational Science and Human Movement (SPPEFF), University of Palermo, 90128 Palermo, Italy

**Keywords:** child neglect, parental competence, maltreatment, child abuse, assessment, negligence

## Abstract

This paper grants some considerations on a critical phenomenon for child health: child neglect. It is an omission-type form of childhood maltreatment, which is widespread but very hard to intercept. For the assessment of child neglect, the Italian Society of Pediatric Psychology (S.I.P.Ped.) has developed and validated a specific assessment technique (the C.N.A. technique). It is supposed to be for parents of children between 3 and 9 years old. It is based on a paradigm that identifies the dysregulation of parental competence as the cause of neglect. It can occur in hypo- or hyperactivation of three fundamental factors (recognition, stimulation, and care). The child neglect assessment technique (C.N.A.) differs from the retrospective tools available in the literature since it allows for interception of the “signs” of possible child neglect when negligence occurs.

## 1. Introduction

The WHO describes “Child Neglect” as “*the failure to provide for the child’s development in all spheres: health, education, emotional development, nutrition, shelter, and safe living conditions, within the context of resources reasonably available to the family or caregivers. Neglect causes or has a high likelihood of causing harm to the child’s physical, mental, spiritual, moral, or social health or development*” [1]. Child neglect as negligence in caring for a child is—nowadays—one of the most widespread forms of child maltreatment.

Although neglect is the most common sort of maltreatment, very little is known about it compared to other types. Research has mainly focused on the study of sexual and physical abuse [2,3].

The scientific literature on this issue has identified specific explanatory theoretical models of child neglect: the “Parental deficit model” [4,5], which identifies the parent with their mental health condition and its characteristics as the cause of child neglect; the “Environmental deficit model” [6,7] which connects this form of maltreatment to environmental and material deprivation; and the “Ecological transactional model” [8] which focuses on the continuous and reciprocal interaction between family characteristics and variables related to the environment.

The scientific literature highlights how prior experience of neglect can upset healthy development and have permanent consequences. For example, the development of brain circuits can be interrupted, affecting how children learn, solve problems, and interact with others if the adult feedback given to children is unpredictable, inappropriate, or simply lacking.

Responsive relationships are fundamental since they affect brain architecture through interactions implying reciprocity of actions. When caregivers are sympathetic and reactive to a baby’s signs and requests, they offer an environment full of back-and-forth practices.

A lack of responsive relationships utterly threatens child development and health. Danger perception triggers a stress response by systems, and excessive activation of those systems can have a toxic outcome on developing brain circuitry [9].

When the shortage of responsive relationships endures, the adverse effects of toxic stress can intensify and worsen the impact of the loss of development opportunities. This harsh influence of neglect on the developing brain highlights why it is so dangerous in the earliest years of life. However, it also shows why early interventions are essential to gain lifelong mental health results and enable the next generation’s rewarding parenting.

This paper aims to describe a new tool developed by the Italian Society of Pediatric Psychology for the early detection of child neglect signs.

## 2. Consequences of Child Neglect

Long-lasting child neglect is often linked to a wider variety of damage than physical and sexual abuse, but it gains little interest in policy and practice [10]. However, studies on children in different contexts have highlighted that severe deprivation or neglect produces negative outputs. For example, it disrupts how children’s brains develop and process information, increasing the risk of behavioral disorders [11]; it modifies biological stress response systems, leading to a greater risk of anxiety, depression, cardiovascular problems, and other chronic health impairments over a person’s lifetime [12]; and it is linked to a significant risk of learning disabilities and poor school achievement [13].

As is well known, a child’s reaction to neglect can have lifelong and even intergenerational influences. Childhood maltreatment can be linked to later physical, psychological, and behavioral consequences and societal costs [14]. However, the implications for each child may differ broadly and are shaped by a mix of elements, including the child’s age and developmental status when the neglect occurs; the type, frequency, duration, and severity of the maltreatment; and the relationship between the child and the perpetrator [15]. Furthermore, children who face neglect are often victims of other harmful practices (e.g., poverty, domestic violence, parental substance use, and parental psychiatric illness), which makes it hard to know the specific outcomes of neglect [16].

## 3. The Struggle for Child Neglect Detection

Cultural beliefs shape “parental ethno-theory” [17,18], the proper cultural way of taking care of a child, thus heading toward dissimilar descriptions of what careless conduct is [19]. The age at which a child may be left alone at home differs between cultures [20]. Hence, parental action could be considered a deprivation of caregiving in one culture but not another [21].

Moreover, different from physical and sexual abuse, the problem of neglect is that there is very little social agreement on when the lack of caregiving becomes so harsh to require the involvement of social services.

Professionals and scholars have endeavored to face this issue by developing classes of neglect that cluster child neglect into wider subtypes. Dubowitz, Pitts, and Black [22] proposed three neglect groups: physical, psychological, and environmental. Similarly, Slack, Holl, Altenberned, McDaniel, and Stevens [23] suggested three subtypes of neglect: physical, psychological, and cognitive, which are somewhat dissimilar to Dubowitz’s. Kantor and her colleagues [24] proposed four classes: emotional, cognitive, supervision, and physical neglect. Erickson and Egeland [25] suggested five types of neglect: physical, emotional, medical, psychological, and educational negligence. So far, there is still no consensus on how many subtypes there should be and which dimensions are involved.

## 4. The Contribution of the Italian Society of Pediatric Psychology

From the perspective of pediatric psychology [26,27,28], child neglect is a form of emergency concerned with the balance between resources and the impairment of a child. Specifically, the Italian Society of Pediatric Psychology (S.I.P.Ped.) considers the omission of parental competence as a fundamental factor of the specific relationships within the family system. These omissive relationships then become factors that define the “nature” of this condition.

The theoretical model developed by S.I.P.Ped. refers to the ecological–transactional model, identifying dysregulation of parental competence as the cause of child neglect, which leads to an omission in intercepting the child’s needs. With dysregulation, we contend with a lack of self-control, low emotional intelligence, a tendency to extremes, poor knowledge of parental maladaptive behaviors by parents, heuristics and bias, an inability to balance behavior, and a lack of prediction relating to consequences of the omissive relationship. Therefore, there is a condition of dysregulation that sees the functions of parental competence altered, namely: the “Scaffolding” [29,30], the “Coping” [31], and the “Caregiving” [32].

According to this model, the omission of adequate responses to the child’s needs can manifest in both the absence and the sense of hypertrophy of that specific behavior. For example, in dysregulated scaffolding, the caregiver can be too assertive or not at all; in dysregulated caregiving, the parent does not consider the child’s thinking, with them being too assertive or not at all. Lastly, regarding dysregulated cognitive coping, the parent can use bias/heuristics in describing the child or not having a role model.

### A Technique for the Early Assessment of Child Neglect: The C.N.A. by the Italian Society of Pediatric Psychology

The S.I.P.Ped. developed the Child Neglect Assessment (C.N.A.) technique to assess child neglect in parents of children aged 3–9 in families of Italian culture. Furthermore, it is a technique that differs from other Italian tools measuring child neglect since they are retrospective, measuring the condition of child neglect in later stages of development. Instead, the C.N.A. detects child neglect as it occurs.

The C.N.A. technique is split into two specific observation tools, which were subjected to a content validation process through the method of judges: (a) a child neglect risk sheet and (b) a coding scheme of the indicators of child neglect. The tools are administered in two different moments using a specific procedure. In such a way, the professional/expert first applies the form on the risk indicators of child neglect. Then, if a score indicates the presence of a real risk, the second tool is administered (the coding scheme on the indicators of child neglect). The teamwork of the S.I.P.Ped. created three ranges of scores to discriminate the risk severity of child neglect by parents: 11–19 low-risk parents, 20–27 medium-risk parents, and 28–36 high-risk parents.

This tool lists the possible risk factors of child neglect; in particular, they are risk factors of parental dysregulation which could lead to child neglect. Expressly, the construction and the validation of this tool have provided for specific elements with the related items: economic resources shortage; the difficulty of the environmental contextualization; the absence of social support; emotional immaturity; psychopathology; addictions; bad mentalized care experiences; and dysfunctional models of representation of the child’s developmental weakness. In addition, the sheet passed through double verification of the validity of the content through the judge’s method. Specifically, it was subjected to a first step of verification of the validity of the content through 65 judges (psychologists), calculating the Fleiss’ K concordance index; in this sense, the tool was changed by modifying/eliminating items that presented weak agreement, adding items, eliminating a factor, which was redundant, and inserting a further factor for the parental deficit model suggested by the judges’ answers: antipathy. Therefore, additional content validation of the new tool form was performed involving another 100 judges (psychologists).

In its final form, the sheet consists of 30 items relating to 8 factors:

1. Psychopathology (e.g., Do you sometimes feel depressed?)
2. Addictions (e.g., Has *it ever happened to you, in very sad moments, to overdo your drinking and/or smoking?*)
3. Inadequate mentalized care experiences (e.g., *Has it occurred to you to rethink the educational styles used by your parents? How do you remember them?*)
4. Emotional immaturity (e.g., *Can you control anxiety under challenging situations involving your child?*)
5. Antipathy (e.g., *Do you often feel dissatisfied with your child?*)
6. Absence of social support (e.g., *Are there people close to you in the most challenging moments?*)
7. Precarity of economic resources (e.g., *Does your family have a single salary?*)
8. Difficulty of environmental contextualization (e.g., *Do you feel comfortable in the neighborhood where you live?*)

As stated, only after using the risk of child neglect sheet can the psychologist apply the coding scheme on child neglect. Then, it was applied during a narrative interview (four open questions) addressed to the parent, constituting an experimental condition. This tool was subjected to two evaluations made by the judges’ method, and the degree of agreement among the evaluators was tested with Fleiss’ K; values of kappa can range from −1.0 to 1.0, with −1.0 indicating perfect disagreement below chance, 0.0 indicating agreement equal to probability, and 1.0 indicating perfect agreement above chance; all values represented by a k ≥ 0.40 were considered acceptable. In its final form, the coding system has 5 factors with 24 items, 4 for each factor; in contrast, factor E (discure) was further divided into hypocure (Ea) with 4 items and hypercure (Eb) with 4 other items. The five factors are:

- Hypo-stimulation (e.g., *The child is not guided in experimenting with new behaviors*)
- Hyper-stimulation (e.g., *The child’s behaviors are totally guided and directed by the parent*)
- Disavowal (e.g., *The thoughts, opinions, intentions, emotions, and desires of the child are not recognized*)
- Adultization (e.g., *The parent asks the child to express opinions that concern adults only*)
- Discure (hypocure: e.g., *The parent does not pay attention to the physical health of the child*; hypercure: e.g., *Attention is continually paid to the hygiene of the child*).

Another crucial methodological step was attributing a “weight” to each of the five factors of child neglect through the involvement of the 45 blinded judges; each judge attributed a value of importance to each item of each factor (from 1 = not very to 3 = very). The average scores for each factor were then evaluated: 1.5 for hypostimulation and 1.5 for hyperstimulation; therefore, the score of each reference item of the two factors was 0.37, 3.5 for adultization and 3.5 for denial; therefore, for each of the reference items of the two factors the score was 0.87, and 8 for the discure (4 for the hypocure and 4 for the hypercure). Therefore, the score of each reference item of the two factors is 1. Based on this assessment of the importance of the factors, a *cut-off* of 10 (half + 1) was set, and we are waiting for data on the standardization.

The technique has some specific strengths: first, it uses tools to intercept the “signs” of possible child neglect when maltreatment occurs, unlike the retrospective tools on child neglect currently used in Italy. It allows for intervention immediately when severe dysregulation of parental competence, such as omissive behavior, is present. Implementing actions to support the child and promote change as soon as possible is essential. A further strength point is a possibility for other professionals, besides the psychologist (social worker, pediatrician, pedagogue, educator, etc.), to use the technique through a specific integrated procedure. It can facilitate more effective action to support parents with the dysregulation of parental competence.

## Data Availability

The data presented in this study are available on request from the corresponding author. The data are not publicly available due to the Italian privacy law.
